# A New Therapeutic Agent in the Treatment of Pulmonary Tuberculosis

**Published:** 1913-06

**Authors:** Sevier Warren

**Affiliations:** San Angelo, Texas


### A New Therapeutic Agent in the Treatment of Pulmonary Tuberculosis.

BY SEVIER WARREN, M. D., M. R. C., SAN ANGELO, TEXAS.

"The production of leukocytosis is, in itself, of little value unless at the same time the amount of opsonin in the blood, the specific opsonic index, is high enough to favor phagocytosis. The simultaneous stimulation of leukocytosis and production of opsonins would, therefore, seem to be the object aimed at in many of the bacterial infections that have so far proved most refractory to serum therapy."—Jordan.

Herein is a statement of fact that stands an impenetrable barrier against any possibility of a serum cure—not only for tuberculosis, but for all bacterial infections that entail prolonged, wasting and cumulative pathological phenomena.

Recognizing this as an impossibility—and it has been repeatedly proven—we must turn from the therapy of organic and biological products to the therapy of inorganic or chemical agents.

Believing this to be not only the correct but the only logical solution whereby a cure might be established I begun, several years ago, a series of experiments with carbolic acid held in suspension in the mineral oil commonly known as petrolatum—which I first used as an inunction, but later hypodermically.

These experiments have proven so amazingly successful and encouraging that I feel that I should give them to the profession, confidently believing that in phenol-petrolatum we have an agent that simultaneously stimulates leukocytosis and the production of opsonins—and in as yet some inexplicable way induces an immunity in the phagocytes against the resultant toxines of bactericidal decomposition and tissue waste.

As we know, phagocytosis may be intensified or diminished in various ways. For instance, the power of leukocytes to destroy micro-organisms is greatly increased by the addition of serum from immunized animals. The activity of the phagocytes is due, according to Wright and other observers, to the presence in the blood and other fluids of certain substances which act in some unknown manner upon bacteria and prepare them for digestion by the phagocytes.

These bacteriotropic substances have been termed opsonins. They claim, and have so demonstrated, that opsonins are also present in the blood of normal animals but can be increased in amount by immunization. It would follow, therefore, that this stimulation is as much if not more of a physiological protest against the introduction of foreign matter into the blood and lymph as it is a result of pathological changes. Indeed, it seems far more logical to assume that these are not bacteriotropic substances, but are, as I believe them to be, radio-active substances that are physiologically present in all sera.

As we all know, the slightest injury to normal tissue, whether it be mechanical, traumatic, or infective, is resented by nature, who at once forwards her host of leukocytes to the point of attack or injury, if it be local, and registers her protest in a general leukocytosis if the infection be general.

While in acute infections, as diphtheria, pneumonia and meningitis, the injection of immunized blood or antitoxins does, as we all concede, result in an increased activity of the phagocytic activity, and that there is an immediate fall in the opsonic index, which, however, gradually rises above normal and subsides again, with the crisis, to below normal. All of which is contrary to the theory that opsonins are bacteriotropic substances, but on the other hand it seems to me is proof positive that this function of phagocytic stimulation is a physiological one and is, I repeat, due possibly to a radio-activity of the blood serum or the haemoglobin of the red cells.

Granting that opsonins are bacteriotropic substances and that they can be materially increased, and phagocytosis stimulated by the introduction of living or dead bacteria or their products, is there any reason or excuse by which we can justify the administration of tuberculin? By way of preface let me say that if tuberculin had not had in a great measure the endorsement of the medical profession—in the past—it is my opinion that today its manufacture and sale would be prohibited by the National Government. While this support and endorsement has been withdrawn by all the leading authorities on the practice of medicine, yet I repeat it should fall under the ban of the law.

Clinically, there has never been one favorable symptom that would excuse its use. Bacteriologically, it convicts itself.

There are no more pathetic pictures than the ones which we see in this western country of those who have sought climatic advantages as the last resort from pulmonary tuberculosis—and tuberculin.

Patients in whom nature has been fighting an acquired condition impeded with the weight of a scientific abortion.

Oftentimes when reproving a patient for abusing his stomach with all of the patent nostrums that are vended I am met with this retort: Well, Doctor, what am I to do? I have taken tuberculin for six months, a year or maybe two years. You see my condition; I am worse than I ever was.

What reply can you make? The reply that will soon be made—to our disgrace—will be found on the Federal statute books.

The most serious phase in the tubercular process—and the one to which we give too little consideration—is that of the complication of mixed infection. The immediate drop in the hæmoglobin is the first microscopic findings that fixed, bacteriologically, the positive diagnosis of pulmonary tuberculosis, the presence of the tubercle bacillus being a matter only of relative importance. I have found a decided diminution in the haemoglobin estimate to exist before the clinical symptoms become of sufficient intensity to cause much physical distress to the patient. I have also observed it to remain at a very low percentage when the patient under rest and forced feeding has added pounds of weight, and where the fever, cough and exhaustive symptoms have almost entirely disappeared. Under these circumstances I still consider the prognosis for an arrested cure as unfavorable.

On the other hand I have noted the haemoglobin to slowly creep upwards without any very apparent changes in the clinical symptoms. And invariably I find the ultimate outcome in these cases more promising.

Hand in hand with a heavy mixed infection we get the low haemoglobin estimate. Why? Because the staphylococci, under certain conditions, produce a substance that acts upon the stroma of the red blood corpuscles in such a way as to cause the dissolving out of the haemoglobin.

Therefore, accepting the haemoglobin estimate as the true bacteriological barometer of the constitutional condition it follows that any mode of treatment, or any therapeutic agent, which increases the haemoglobin is a logical and sensible treatment. Nature, herself, attempts to point this out to us in demanding pure air, quantities of easily assimilated food, and physiological and mechanical rest.

I believe that I am warranted in saying that nature would effect a cure in most if not all cases along these practical lines if we could eliminate the mixed infection. The fact that the staphylococci are continually depleting her haemoglobin handicaps her to the extent that she cannot furnish sufficient oxygen to sustain her vital equilibrium. It is my opinion that it is this deficiency of oxygen in the blood that prevents her bringing into action the forces with which she is endowed to repel the invasion of all pathogenic bacteria—a physiological function that both stimulates her leukocytes to phagocytic action and the power to immunize herself against the cumulative toxins arising from the debris of tissue waste and bacteria.

What is the therapeutic rationale that stimulates nature's method of striving for a cure in pulmonary tuberculosis?

Proceeding upon the theory that nature not only in animal but in plant life oftentimes succeeds in attaining spontaneous cures we must concede that fact that she acquires an immunity or an immunizing function either from the elements of her makeup or from the selective powers that she has of abstracting from the elements and food all that is necessary to sustain normal life as well as to recruit from her defensive forces.

The old saying of *"similia similibus curantur"* does in no way apply to an acquired immunity nor does the theory of "familiarity (with infection) breeds contempt." To the contrary, nature proceeds to acquire this immunity and to conduct her campaign against this invasion of pathogenic bacteria in the most scientific manner.

Besieged as she is from the moment of conception to the ultimacy of death by all the forces of dissolution and destruction she not only wages incessant war against an attacking force, but must care for the dead of her enemies as well as her own.

This is most remarkedly typified in her fight against tuberculosis. She is being attacked, first, by the invading host of bacilli, staphylococci, and streptococci. Secondly, she is being constantly infected from the decomposition of the dead bacteria, her own dead and disintegrating leukocytes. Finally, she is being constantly called upon to recruit her own forces. To meet these exigencies she has—impoverished blood, usually less than two-thirds of a normal haemoglobin, a lymphatic system riddled with tubercular foci—her lungs, kidneys, intestinal tract, in fact, all of her excretory organs involved much below normal in their functional activity.

What does she do? What can she do?

She calls per-emptorily for food to replenish her haemoglobin and to recuperate her blood-making organs. She sends mandatory directions to every atom of tissue commanding absolute rest, and she calls upon that function with which she is endowed to give her more immunized leukocytes, but this function as we believe is wholly dependent upon the presence of an unimpaired haemoglobin, and herein as I believe lies the key to the situation.

Aid nature to overcome or to lessen the intensity of the mixed infection and if you have not given her a cure you have so materially aided her in the fight that she is making what I am confident that with the aid of common sense treatment such as proper food, absolute rest and hydrotherapy, she will effect a complete cure in practically every case.

What therapeutic agent will control or dissipate a mixed infection, and how?

Phenol-petrolatum clinically and bacteriologically demonstrates its efficacy.

When administered hypodermically into the deep tissues it is taken up by the lymphatic system and consequently conveyed directly to the blood-making organs, where it not only stimulates leukocytes to phagocytic activity, but is either itself bactericidal or induces opsonic action in so far as the cocci are concerned. This is demonstrated by an immediate increase in the haemoglobin, a subsiding of febrile conditions and later dimunition in the staphylococci and streptococci as seen in the microscopic field.

To any one wishing to demonstrate these facts for himself I would suggest that he have his chemist prepare a one-half per cent strength of phenol-petrolatum and give, first, an initial dose of fifteen minims into the interscapular tissues, following with the minimum dose of two minims, which is increase one minimum daily up to physiological tolerance. I would be glad to receive your reports.—*Charlotte Medical Journal.*

